# Correction to “Decoding the Spatiotemporal Dynamics of Neural Response Similarity in Auditory Processing: A Multivariate Analysis Based on OPM‐MEG”

**DOI:** 10.1002/hbm.70228

**Published:** 2025-05-08

Liu, C., Y. Ma, X. Liang, M. Xiang, H. Wu, and X. Ning. 2025. “Decoding the Spatiotemporal Dynamics of Neural Response Similarity in Auditory Processing: A Multivariate Analysis Based on OPM‐MEG.” *Human Brain Mapping* 46, no. 4: e70175. https://doi.org/10.1002/hbm.70175.

One of the corresponding author's name was inadvertently misspelled as “Xiaoling Ning”. It should be corrected to “Xiaolin Ning”.

The online version of the article has been corrected accordingly.

We apologize for this error.

